# Supplementary material for: Real-time photonic blind interference cancellation
Source: Nat Commun. 2023 Dec 11;14:8197. doi: 10.1038/s41467-023-43982-w (PMC10713617; doi:10.1038/s41467-023-43982-w)
Supplement: Supplementary file 1 — Supplementary Information [file 41467_2023_43982_MOESM1_ESM.pdf]

Real-Time Photonic Blind Interference Cancellation

Joshua C. Lederman, Weipeng Zhang, Thomas Ferreira de Lima, Eric C. Blow, Simon Bilodeau, Bhavin J. Shastri, Paul R. Prucnal

Supplementary Notes

The Photonic Transfer Function

The transfer function T of a micro-ring resonator with Q-factor Q and center frequency ω_0 may accurately be approximated as a Lorentzian [1]:

$$T(\delta) = \frac{1}{1+\delta^2} \quad \delta = \frac{Q}{\omega_0}(\omega - \omega_0) \tag{1}$$

Weight setting is performed by applying a current i to a resistive heater near the micro-ring, producing a temperature shift ΔT:

$$\Delta T \propto i^2 \rightarrow \Delta T = \alpha i^2 \tag{2}$$

Notably, the specific nonlinear relationship of temperature to current aids the nonlinearity, and the properties of PN-junction micro-rings would vary.

The temperature shift induces a slight index of refraction shift due to the thermo-optic effect in silicon, which in turn slightly shifts ω_0. Each of these shifts are sufficiently small that their associated functions may be approximated as linear. With ω_{00} represented the center frequency at the ambient temperature:

$$\omega_0 \approx \omega_{00} + \beta \Delta T \quad \beta = \frac{d\omega_0}{dT} \tag{3}$$

Recognizing $|\omega - \omega_0| \ll \omega_0$ and $|\omega - \omega_{00}| \ll \omega_{00}$, we combine Eqs. 1, 2, and 3:

$$\delta \approx \frac{Q}{\omega_{00}}(\omega - \omega_0) = \frac{Q}{\omega_{00}}\left(\omega - \omega_{00} + \alpha\beta i^2\right) = a + bi^2 \tag{4}$$

$$a \equiv \frac{Q}{\omega_{00}}(\omega - \omega_{00}) \quad b \equiv \frac{Q}{\omega_{00}}\alpha\beta \tag{5}$$

$$T(i) = \frac{1}{1 + (a + bi^2)^2} \tag{6}$$

Under an idealized model of the balanced broadcast-and-weight filter, all transmitted light generates negative photocurrent with responsivity R, and all other light generates positive photocurrent with the same responsivity. Hence, total photocurrent I follows:

$$I(i) = R\left(-T(i) + (1 - T(i))\right) = R\left(1 - 2T(i)\right) \tag{7}$$

Eq. 7 represents the photonic transfer function.

We seek to examine the linearity of the transfer function about $I(i) = 0$. The second derivative $I''(i)$ represents a simple proxy for linearity. The second-derivative formula is fairly complex, but it simplifies greatly when evaluated at i_0:

$$I(i_0) \equiv 0 \quad i_0 = \sqrt{\frac{1-a}{b}} \quad I''(i_0) = 2R(2a-1)b \tag{8}$$

While R and b are fixed by the photonic system design, a may be tuned by selecting the appropriate ω. Selecting a to maximize the linearity at the zero-weight point:

$$I''(i_0) = 0 \rightarrow a = \frac{1}{2} \rightarrow \tag{9}$$

$$\omega = \omega_{00}\left(1 + \frac{1}{2Q}\right) = \omega_{00} + \frac{1}{4}\omega_{\mathrm{FWHM}} \tag{10}$$

Where ω_{FWHM} represents the full-width at half-maximum of the transfer function T. The optimal operating frequency for micro-ring linearity about the zero-weight point is slightly offset from the resonance peak, a more conventional operating point. As micro-rings typically have narrow resonances and high quality factors, most tunable lasers have a range easily sufficient to set the operating wavelength at the target point.

In practice, the photonic transfer function is more complex, with additional terms corresponding to micro-ring absorption, differing photodetector responsivities, and the dependence of heater resistance on temperature. Nevertheless, experimental measurements of the photonic transfer function confirm the high level of linearity at the zero-weight point, as shown in the manuscript.

Derivation of Variance Minimum at Origin

The variance of a measured signal $m(t)$ is defined as follows:

$$\sigma^2 = \int_{-\infty}^{\infty} m^2(t)dt \tag{11}$$

The measured signal is a function of the source signals $\mathbf{s}(t)$, weights $\mathbf{w}$, and mixing matrix $\mathbf{M}$:

$$m(t) = \mathbf{w} \cdot \mathbf{r}(t) \quad \mathbf{r}(t) = \mathbf{M}\mathbf{s}(t) \tag{12}$$

The source signals are independent, and therefore have a unit covariance matrix. With the normalization factors pulled into the matrix $\mathbf{M}$ for the duration of this proof:

$$\int_{-\infty}^{\infty} s_i(t)s_j(t)dt = \delta_{ij} \tag{13}$$

We assume distinct mixing proportions for each signal mixture—that the mixing matrix $\mathbf{M}$ is linearly independent:

$$|\mathbf{M}| \neq 0 \tag{14}$$

All integrals below are implied to have infinite bounds.

Calculating the gradient of σ^2:

$$\nabla_{\mathbf{w}}\sigma^2 = \nabla_{\mathbf{w}} \int (\mathbf{w} \cdot \mathbf{r}(t))^2 \, dt \tag{15}$$

$$= \int \nabla_{\mathbf{w}} (\mathbf{w} \cdot \mathbf{r}(t))^2 \, dt \tag{16}$$

$$= 2 \int (\mathbf{w} \cdot \mathbf{r}(t)) \, \mathbf{r} dt \tag{17}$$

Shifting to subscript-summation notation:

$$\frac{\partial \sigma^2}{\partial w_j} = 2 \int w_i r_i(t) r_j(t) dt \tag{18}$$

$$= 2 \int w_i M_{ik} s_k(t) M_{jl} s_l(t) dt \tag{19}$$

$$= 2 w_i M_{ik} M_{jl} \int s_k(t) s_l(t) dt \tag{20}$$

$$= 2 w_i M_{ik} M_{jl} \delta_{kl} \tag{21}$$

$$= 2 w_i M_{il} M_{jl} \rightarrow \tag{22}$$

$$\nabla_{\mathbf{w}}\sigma^2 = 2\mathbf{M}\mathbf{M}^T w \tag{23}$$

Optima of σ^2 with respect to $\mathbf{w}$ may only exist when the gradient is zero:

$$\nabla_{\mathbf{w}}\sigma^2 = 2\mathbf{M}\mathbf{M}^T\mathbf{w} = \mathbf{0} \tag{24}$$

$$\left|\mathbf{M}\mathbf{M}^T\right| = 0 \quad \text{or} \quad \mathbf{w} = \mathbf{0} \tag{25}$$

$$|\mathbf{M}| \neq 0 \rightarrow |\mathbf{M}|^2 = \left|\mathbf{M}\mathbf{M}^T\right| \neq 0 \rightarrow \mathbf{w} = \mathbf{0} \tag{26}$$

Optima may only exist at the single point where $\mathbf{w} = \mathbf{0}$. Furthermore:

$$\frac{\partial \sigma^2}{\partial w_i \partial w_j} = 2 M_{ik} M_{jk} \rightarrow \mathbf{H}_{\sigma^2} = 2MM^T \tag{27}$$

Where $\mathbf{H}_{\sigma^2}$ represents the Hessian matrix of σ^2 with respect to $\mathbf{w}$.

$$|\mathbf{H}_{\sigma^2}| = 2\,|\mathbf{M}|^2 \quad |\mathbf{M}| \neq 0 \rightarrow |\mathbf{H}_{\sigma^2}| > 0 \tag{28}$$

The variance is uniformly convex. Therefore there exists a single local minimum of σ^2 at $\mathbf{w} = \mathbf{0}$.

Statistic Mathematics

It is possible to mathematically model the uncertainty of the variance estimator in order to gain a deeper understanding of the relationship between sampling parameters and statistic uncertainty, and we do so here in order to provide additional context for our results.

The uncertainty γ of the variance estimator S^2 may be defined:

$$\gamma^2 = \mathbb{E}\left[\left(S^2 - \mathbb{E}\left[S^2\right]\right)^2\right] \tag{29}$$

It may be approximated with $m \gg 1$ samples of S^2:

$$\gamma^2 \approx \frac{1}{m-1}\sum_{i=1}^{m}\left(S_i^2 - \frac{1}{m}\sum_{j=1}^{m} S_j^2\right)^2 \tag{30}$$

If the signal samples were randomly chosen in time and therefore independent and identically distributed (IID), there would be a fixed relationship between the uncertainty of the variance and the underlying signal variance σ^2 and kurtosis κ:

$$\gamma^2 = \frac{\sigma^4}{n_s}\left(\kappa - 1 + \frac{2}{n_s - 1}\right) \approx \frac{S^4}{n_s}\left(K - 1 + \frac{2}{n_s - 1}\right) \tag{31}$$

Where n_s represents the number of samples used to calculate the estimator. Note that for large n:

$$\gamma \propto \frac{1}{\sqrt{n_s}} \tag{32}$$

Though the samples are periodic and therefore not IID, Eq. 32 nevertheless describes in general terms the increase in measurement consistency observed as n increases.

Supplementary Fig. 1 shows the variance estimator data of Fig. 5b-c with the addition of predicted estimator uncertainties according to Eq. 31, assuming random sampling. For most sampling parameters periodic sampling meaningfully outperforms random sampling, with lower levels of statistic uncertainty compared to random sampling. This is likely owed to the more even distribution over time of periodic samples as compared to random ones. However, periodic sampling may produce very poor behavior when the sampling rate matches too closely to underlying signal frequency components. Samples would be chosen selectively from some parts of the signal distribution depending on the exact sampling start time, greatly increasing the measurement variability and therefore uncertainty. Moreover, periodic sampling also performs poorly when there are signal frequency components with a period comparable to that of the total sampling duration. Sampling that occurs predominantly during a fast-changing portion of the signal (e.g. near the zero-point of a sinusoid) would measure distinct statistics as compared to sampling occurring during a slow-changing portion of the signal (e.g. near the peak of a sinusoid). Care must be taken to choose sampling durations that are best suited to the RF environment.

During experimental measurements a 1,137-bit random repeating 200 MBaud RF signal was generated for separation, with a length limited by the AWG storage capacity. This 5.68 µs periodic signal degrades statistic measurement consistency where the measurement time has a comparable period. When taking 2,048 samples per measurement this artifact appears at high sampling rates between approximately 500 MSPS and 2 GSPS, accounting for the sharp increase in uncertainty of periodic sampling in comparison to random sampling at this portion of Supplementary Fig. 1. When sampling at 7.68 MSPS this artifact occurs at a very low samples per measurement and is therefore obscured by the naturally higher uncertainty of such measurements. The kurtosis data shows similar behavior and may be explained in the same manner. Such artifacts result from the experimental setup and would not be observed using aperiodic bitstream data.

Matrix Ill-Condition Numbers

We seek a metric to quantify the difficulty of performing effective interference cancellation for a given mixing scenario. Specifically, this metric should quantify the sensitivity of the demixed output signal to errors in the cancellation weights. Mixing matrices where the demixed outputs are highly sensitive to errors in the cancellation weights are more challenging to demix, as the cancellation weights must be determined with a greater level of precision.

Mathematically, this metric is the matrix ill-condition number $\alpha(\mathbf{M})$, defined as follows, using the L^2 norm [2]:

$$\alpha(\mathbf{M}) = \|\mathbf{M}\| \cdot \left\|\mathbf{M}^{-1}\right\| \tag{33}$$

The higher the ill-condition number, the greater the sensitivity of the demixed output signals to errors in the cancellation weights and the more challenging it is to perform weight identification.

We can evaluate the ill-condition numbers of our two experimental test matrices:

$$\mathbf{M}_1 = \begin{pmatrix} 0.6 & 0.4 \\ 0.4 & 0.6 \end{pmatrix} \quad \mathbf{M}_2 = \begin{pmatrix} 1 & 0.5 \\ 1 & 0.2 \end{pmatrix} \tag{34}$$

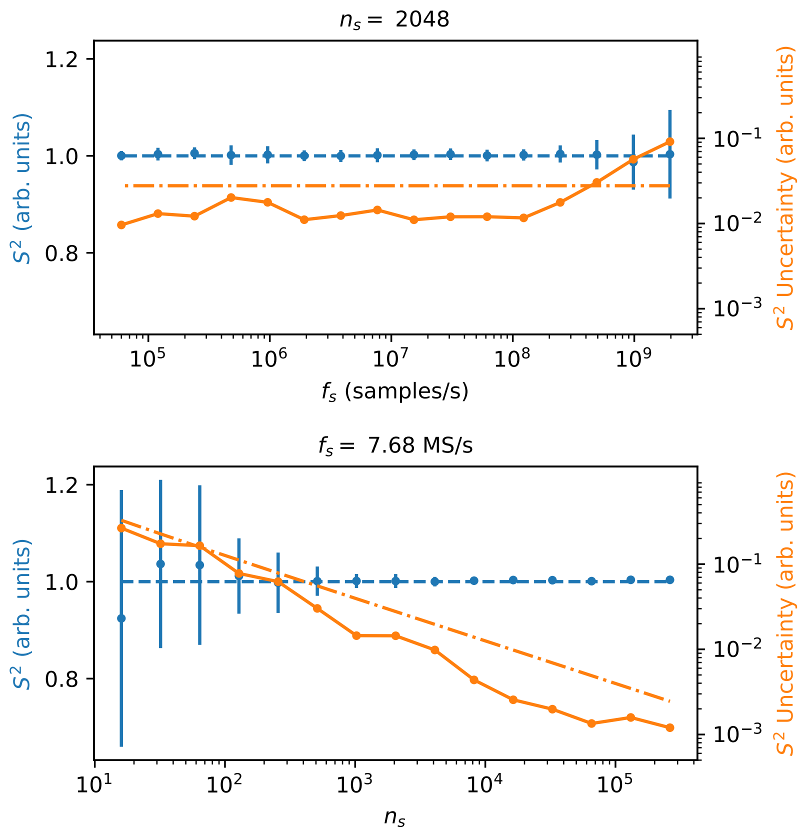

Supplementary Fig. 1. S^2 consistency measurements. This data matches that shown in Fig. 5b-c with the addition of dot-dash orange lines representing the theoretical S^2 uncertainties expected if the collected samples were randomly chosen, following to Eq. 31.

SUPPLEMENTARY TABLE I
ADC Power Consumption Calculation

Role	b (bits)	SNDR (dB)	f_s (GS/s)	$\mathrm{FoM_S}$ (dB/J)	P (µW)
Digital Electronic System Digitization	6	37.9	2	164	244
Digital Electronic System Digitization	6	37.9	10	150	30,700
Photonic System Demodulation	1	7.78	2	164	0.239
Photonic System Demodulation	1	7.78	10	150	30.0
Photonic System Statistic Calculation	6	37.9	0.1	176	0.771

$$\alpha(\mathbf{M}_1) = 5 \quad \alpha(\mathbf{M}_2) = 7.5 \tag{35}$$

$\mathbf{M}_2$, with its higher ill-condition number, represents a more challenging demixing problem, accounting for the decreased level of kurtosis uncertainty required to reliably demix the signal.

Power Analysis

We estimate ADC power consumption by assuming that, for a given ADC technology, the relationship between an ADC's bit precision b, power consumption P, and sampling rate f_s follows the formula of the Schreier figure of merit ($\mathrm{FoM_S}$):

$$P = \frac{f_s}{2} 10^{\frac{1}{10}(\mathrm{SNDR} - \mathrm{FoM_S}(f_s))} \tag{36}$$

SNDR, the signal to noise plus distortion ratio, relates to b as follows:

$$\mathrm{SNDR} = 6.02 \text{ dB} \cdot b + 1.76 \text{ dB} \tag{37}$$

The $\mathrm{FoM_S}$ for each f_s is drawn from recent reports, as documented in the manuscript. Note that both the digitization ADCs of the digital electronic system and the demodulation ADCs of the photonic system scale in sampling rate with the incoming signal bandwidth, but the statistic calculation ADCs do not, due to the application of sub-Nyquist sampling. Supplementary Table I reports on the calculation.

SUPPLEMENTARY TABLE II
DIGITAL PROCESSING POWER CONSUMPTION CALCULATION

Role	Incoming Signal Rate (GS/s)	Power (μW)
Digital Signal Recovery	2	2,000
Digital Signal Recovery	2	10,000
Statistic Calculation	0.1	100

Digital processing power consumption is estimated by assuming a fixed 1 pJ/MAC energy requirement. Power consumption is calculated per unit incoming signal per MAC, such that the power consumption is the product of the incoming signal rate and the 1 pJ/MAC energy consumption, as shown in Supplementary Table II.

Power is required in the photonic sub-system to generate the optical carriers and to tune the MRRs. The optical carriers must be sufficiently powerful to guaranty 6-bits precision cancellation despite shot noise, the dominant noise source at this power level. Noise scales linearly with signal bandwidth. As reported in the manuscript, 96 μW/GHz per carrier is required. MRR tuning power required, assuming post-fabrication trimming, is 120 μW, independent of bandwidth.

Supplementary Methods

ICA and PCA for PBSS

Independent component analysis (ICA) and principal component analysis (PCA) represents the primary tools used to perform PBIC.

ICA relies on the fact that, according to the Central Limit Theorem, linear mixtures of independent signals will, in general, have a distribution of values that more closely matches a Gaussian distribution than the source signals. By maximizing the non-Gaussianity of a mixture, one can identify the underlying sources of that mixture.

One straightforward measure of Gaussianity is excess kurtosis, referred to here as the kurtosis. The kurtosis of a Gaussian distribution is exactly 0, while other distributions may have higher or lower kurtoses. Sinusoids, in particular, have a kurtosis of -1.5, and it follows that RF signals, which are typically constructed of sine-like constituents, tend to have below-Gaussian kurtoses. Therefore, choosing $\mathbf{w}$ to minimize the kurtosis of $m(t)$ ensures $m(t)$ corresponds to one of the source signals $s_i(t)$.

There are multiple $\mathbf{w}$ corresponding to local kurtosis minima, each one associated with one source signal. Standard mathematical minimization techniques can find one such weight vector in the vast majority of cases, but identifying all local kurtosis minima, regardless of their position, remains a challenge. For this we turn to PCA. In this formulation, the principal components of $m(t)$ with respect to $\mathbf{w}$, labeled $\mathbf{w}_{\mathrm{PC}i}$, correspond to the directions of maximum variance of $m(t)$, with the constraint that each subsequent component must be orthogonal to all previous components:

$$\begin{aligned}
\mathbf{w}_{\mathrm{PC1}} &= \underset{\mathbf{w};\ \|\mathbf{w}\|=1}{\arg\max}\ \sigma^2 \\
\mathbf{w}_{\mathrm{PC2}} &= \underset{\mathbf{w};\ \|\mathbf{w}\|=1;\ \mathbf{w}\perp\mathbf{w}_{\mathrm{PC1}}}{\arg\max}\ \sigma^2 \\
&\vdots
\end{aligned} \tag{38}$$

The principal components and their associated variances may be applied to construct a *whitening matrix* $\mathbf{W}_{\mathrm{PC}}$ [3]. The whitening matrix describes a transformation to a new weight coordinate basis, denoted with $'$:

$$\mathbf{w}' = \mathbf{W}_{\mathrm{PC}}^{-1}\mathbf{w} \tag{39}$$

$m(t)$ in the new basis has a unique property: weight vectors corresponding to the independent source signals are orthogonal. We can now solve the problem posed earlier; once we find one independent component by minimizing kurtosis we can then run a further kurtosis minimization under the constraint that the weight vector must be orthogonal to that of the original independent component. ICA may be completed in a process analogous to independent component extraction:

$$\begin{aligned}
\mathbf{w}'_{\mathrm{IC1}} &= \underset{\mathbf{w}';\ \|\mathbf{w}'\|=1}{\arg\min}\ \kappa \\
\mathbf{w}'_{\mathrm{IC2}} &= \underset{\mathbf{w}';\ \|\mathbf{w}'\|=1;\ \mathbf{w}'\perp\mathbf{w}'_{\mathrm{IC1}}}{\arg\min}\ \kappa \\
&\vdots
\end{aligned} \tag{40}$$

The cancellation vector associated with source signal i, $\mathbf{c}_i$ follows:

$$\mathbf{c}_i = \mathbf{W}_{\mathrm{PC}}\mathbf{w}'_{\mathrm{IC}i} \tag{41}$$

Supplementary References

[1] A. N. Tait, M. A. Nahmias, B. J. Shastri, and P. R. Prucnal, “Broadcast and weight: An integrated network for scalable photonic spike processing,” *J. Lightwave Technol.*, vol. 32, no. 21, pp. 3427–3439, Nov 2014.

[2] W. Zhang, A. Tait, C. Huang, T. Ferreira de Lima, S. Bilodeau, E. C. Blow, A. Jha, B. J. Shastri, and P. Prucnal, “Broadband physical layer cognitive radio with an integrated photonic processor for blind source separation,” *Nature Communications*, vol. 14, no. 1, p. 1107, Feb 2023.

[3] C. M. Bishop and N. M. Nasrabadi, *Pattern recognition and machine learning*. Springer, 2006, vol. 4, no. 4.
